# Late-onset paradoxical reactions 10 years after treatment for tuberculous meningitis in an HIV-negative patient: a case report

**DOI:** 10.1186/s12879-018-3229-z

**Published:** 2018-07-06

**Authors:** Akira Machida, Tasuku Ishihara, Eiichiro Amano, Shinichi Otsu

**Affiliations:** Department of Neurology, Tsuchiura Kyodo General Hospital, 4-1-1Otsuno, Tsuchiura-shi, Ibaraki, 300-0028 Japan

**Keywords:** Tuberculous meningitis, Paradoxical reactions, Tuberculoma

## Abstract

**Background:**

Although paradoxical reactions (PRs) to anti-tuberculosis (anti-TB) therapy during treatment are well-established occurrences, PRs presenting as a new lesion after the completion of treatment are extremely rare, and little is known about the management of such cases, particularly of central nervous system (CNS) tuberculosis.

**Case presentation:**

A 27-year-old female, with a past medical history of tuberculous meningitis 10 years ago and who completed the anti-TB treatment with asymptomatic remnant tuberculomas in the basal cistern, was admitted to our hospital because of a headache and the worsening of pre-existing visual disturbance. Contrast-enhanced T1-weighted brain magnetic resonance imaging (MRI) revealed new tuberculomas in the left sylvian fissure with a diffuse low signal around it. Because repeated polymerase chain reaction and *Mycobacterium tuberculosis* culture presented negative results and the patient had no laboratory data suggestive of a relapse of tuberculous meningitis, she was diagnosed with late-onset post-treatment PRs and treated with oral corticosteroids, tapered off over 1 year. Eventually, the symptoms were relieved, and the tuberculomas disappeared.

**Conclusions:**

Clinicians should consider the possibility of PRs long after the completion of tuberculous meningitis treatment. Hence, a precise MRI-based examination is imperative for the follow-up of CNS tuberculosis, and the unnecessary administration of anti-TB drugs should be avoided. The use of corticosteroids as a treatment option for post-treatment PRs is seemingly safe when the isolated *M. tuberculosis* is sensitive to the first-line anti-TB therapy.

## Background

Paradoxical reactions (PRs) to anti-tuberculosis (anti-TB) drugs are defined as the worsening of pre-existing tuberculous lesions or the appearance of new tuberculous lesions in patients whose clinical symptoms initially improved with anti-TB treatment [1–6]. PRs have been reported in approximately one-third of patients with tuberculous meningitis and typically present within the first few months of the anti-TB treatment [4, 5, 7]. In some patients, however, PRs present at a later stage, rendering them difficult to distinguish from treatment failure or tuberculous meningitis relapse. Hence, elucidating the clinical characteristics of PRs and learning how to deal with late-onset PRs constitute an essential aspect of the management of central nervous system (CNS) tuberculosis. Here, we report a case of late-onset post-treatment PRs reported after 10 years of treatment for tuberculous meningitis in an HIV-negative patient.

## Case presentation

**Fig. 1:**
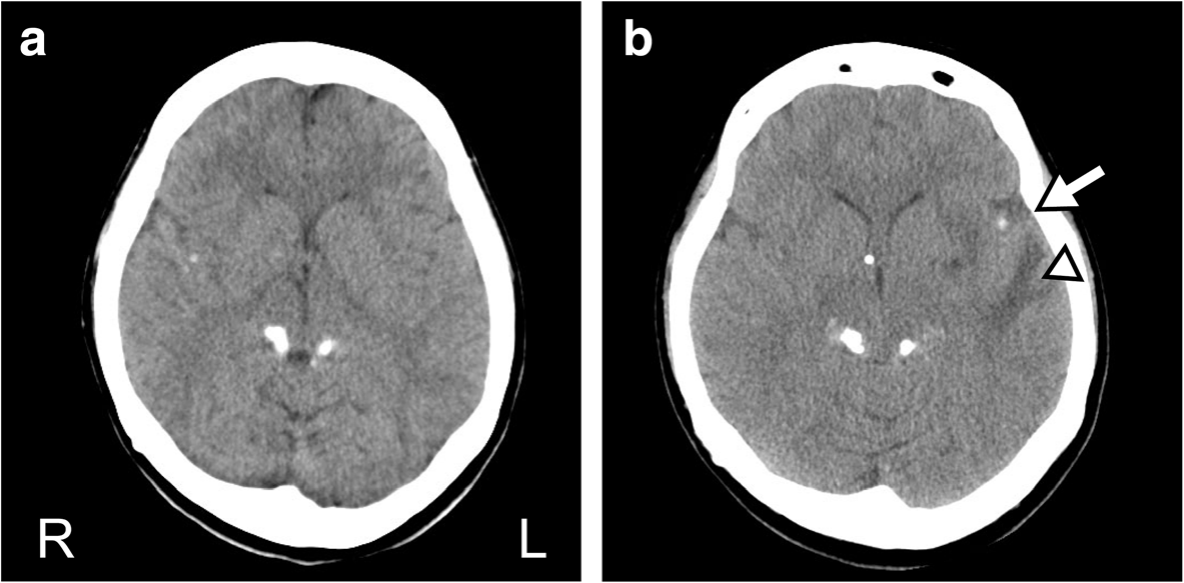
A head CT before and after the onset of PR. **a**, An axial head CT performed just after the completion of anti-TB therapy 10 years ago. Tuberculomas are visible in the bilateral ambient cistern. **b**, An axial head CT performed on admission. New emerging tuberculomas are visible in the left sylvian fissure (arrow), which are accompanied by a low-density area in the left temporal lobe (arrowhead)

A 27-year-old non-HIV-infected female was admitted to our hospital because of a headache and the worsening of pre-existing visual disturbance. Her medical history comprised tuberculous meningitis treated with anti-TB drugs (2-month treatment with isoniazid, 300 mg/day; rifampin, 450 mg/day; ethambutol, 1000 mg/day; and pyrazinamide, 1500 mg/day, followed by a 10-month treatment with isoniazid and rifampin) with adjunctive corticosteroid 10 years ago. The *Mycobacterium tuberculosis* culture was susceptible to all anti-TB drugs, and the patient attained bacteriological remission with a sequela of visual impairment (bilateral counting finger) because of hydrocephalus that was treated with the placement of a ventriculoperitoneal (VP) shunt. Although asymptomatic remnant tuberculomas were reported primarily in the bilateral ambient cistern after the completion of the anti-TB treatment (Fig. 1a), her symptoms remained stable, and she visited a neurosurgical clinic regularly to check the patency of the VP shunt.

**Fig. 2:**
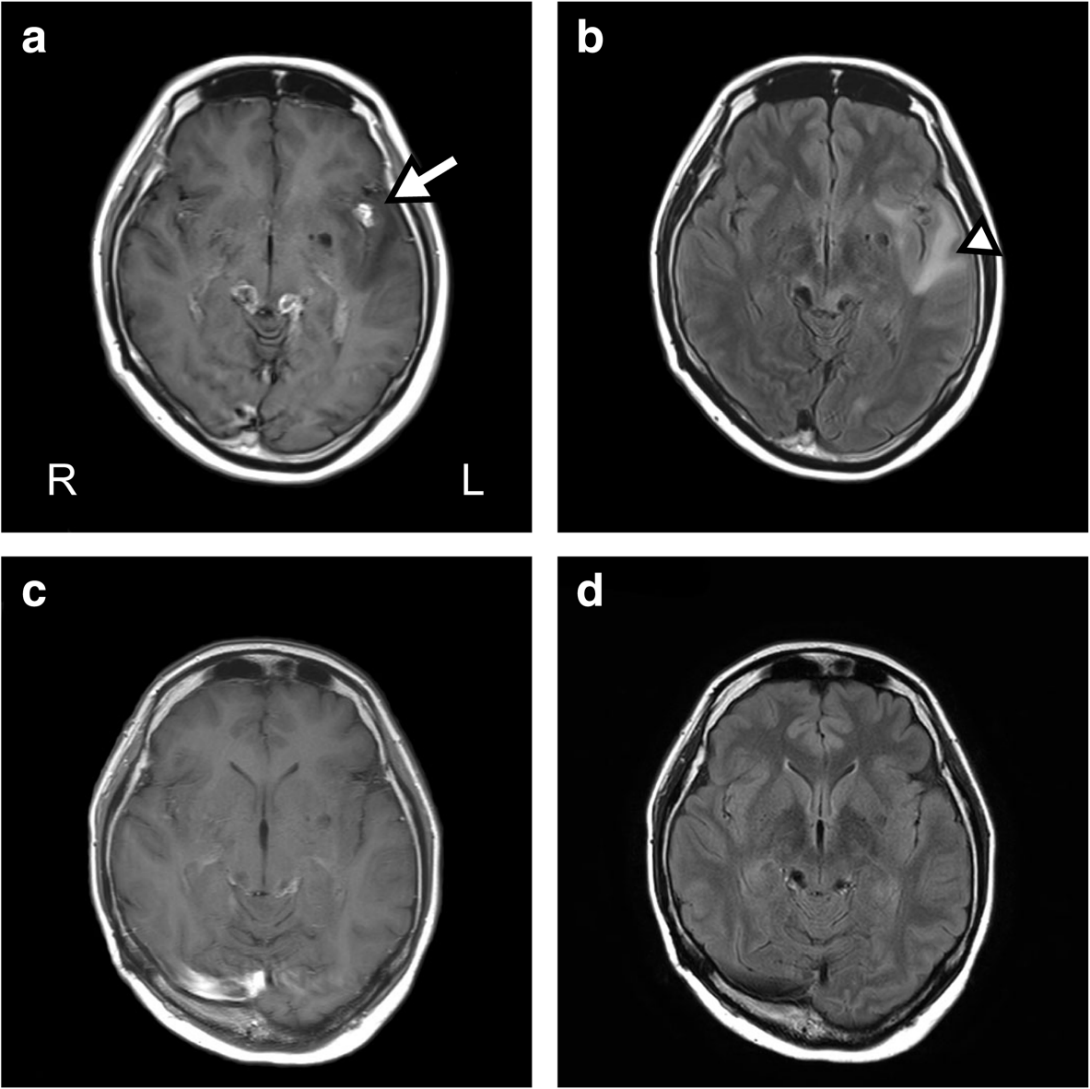
Temopral changes of the brain MRI after PR. **a**, Contrast-enhanced axial T1-weighted brain MRI revealed tuberculomas (arrow) with a diffuse hypointense area around the left sylvian fissure. **b**, Brain FLAIR imaging indicated a hyperintense area around the tuberculomas in the left syluvian fissure, which suggests edematous changes (arrowhead). **c** and **d**, After 3 months of the corticosteroid treatment, the tuberculomas disappeared on contrast-enhanced axial T1-weighted brain MRI, and edematous brain changes around the left sylvian fissure vanished in brain FLAIR imaging

Upon admission, the patient was alert and afebrile, and the neurological examination revealed only the worsening of pre-existing visual disturbance from counting finger to light perception without meningeal irritation. The blood analysis revealed a slight elevation in CRP levels (0.37 mg/dL), and the interferon-γ release assay (T-SPOT) was positive. A chest X-ray revealed no abnormal lesions suggestive of pulmonary tuberculosis. In addition, the cerebrospinal fluid (CSF) testing revealed elevated protein levels (570 mg/dL), no pleocytosis (6/μL), and normal glucose levels (60 mg/dL), with a standard opening pressure (130 mmH_2_O). Notably, antigens of *Cryptococcus*, *Toxoplasma*, and *Aspergillus* in the CSF and the serum antibody against *Taenia solium* were all negative, and no elevation of soluble interleukin-2 receptor and the angiotensin-converting enzyme was observed in the CSF. Furthermore, a head CT revealed new emerging tuberculomas in the left sylvian fissure, which was accompanied by a low-density area in the left temporal lobe (Fig. 1b). While a contrast-enhanced axial T1-weighted brain magnetic resonance imaging (MRI) scan revealed tuberculomas with a diffuse hypointense area around the left sylvian fissure, brain fluid-attenuated inversion recovery (FLAIR) imaging indicated a hyperintense area around the tuberculomas in the left sylvian fissure, suggesting edematous changes (Fig. 2a and b). Repeated mycobacterial culture and real-time polymerase chain reaction (PCR) in the CSF did not indicate the existence of the reactivation of CNS tuberculosis. During these 2 weeks of examination, the symptoms did not worsen without specific treatment. Based on these findings, we diagnosed these lesions as late-onset post-treatment PRs. Accordingly, we prescribed the oral administration of corticosteroids (prednisolone, 30 mg/day, 0.6 mg/kg/day), which were gradually tapered off. After 3 months of the corticosteroid treatment, the tuberculomas disappeared, including those previously present in the bilateral ambient cistern, and brain edematous changes around the left sylvian fissure vanished (Fig. 2c and d). Four months after the onset of PRs, we reduced the dosage of prednisolone to 5 mg/day; however, because a tuberculoma reappeared in the same place of the left sylvian fissure, we increased the dosage of oral prednisolone to 20 mg/day. This time, we carefully tapered off prednisolone over 1 year, and the corticosteroid treatment erased the residual tuberculoma again with no recurrence of tuberculous meningitis. The patient’s symptoms remained stable during 1 year of follow-up without additional anti-TB drugs.

## Discussion and conclusions

Reportedly, PRs represent a delayed-type hypersensitivity reaction secondary to the massive release of destroyed mycobacterial proteins, leading to an uncontrolled inflammatory response, such as the presence of exudates, hydrocephalus, tuberculoma, edema, vasculitis, and infarction [3, 5, 6, 8, 9]. Various studies have reported the onset of PRs as early as 2 weeks and as late as 3 years [3, 5, 7, 8]. To the best of our knowledge, this is the first reported case of the most extended duration from the initiation of the treatment with anti-TB drugs to the onset of PRs.

In our case, we diagnosed the lesions as late-onset post-treatment PRs because the repeated PCR and mycobacterial culture tested negative and no laboratory data suggested a CNS infection. Besides, the patient’s symptoms did not get worse during the 2-week hospitalization without anti-TB drugs, and no recurrence of tuberculous meningitis was reported for 1 year under the corticosteroid treatment without anti-TB drugs. Although calcified granulomas with perilesional edema in the brain are sometimes observed in the case of neurocysticercosis [10–12], the patient had no family history and had never visited or lived in neurocysticercosis endemic areas. In addition, we confirmed the negative results of ELISA-based antibody detection tests for *T. solium*. Therefore, we finally diagnosed the lesions as late-onset post-treatment PRs. Of note, we must distinguish PRs from treatment failure, the relapse of tuberculous meningitis, and other differential diagnosis and refrain from the unnecessary administration of anti-TB or antihelminthic drugs.

The remarkable feature of our case was that PRs had not occurred for 10 years after the completion of anti-TB drugs. A literature review indicated a high propensity of PRs for CNS involvement in comparison to other sites [5, 13], and the time of onset of PRs in CNS tuberculosis appears to be longer than the time of onset at other sites [1, 13]. These findings may be attributed to frequent brain MRI and/or CT scans conducted for patients with CNS lesions, considering the nature of these lesions, which are likely to result in the manifestation of symptoms leading to disease progression. Reportedly, a majority of post-treatment tuberculous lymph node enlargements resolve spontaneously without further anti-TB treatment [14], and the recurrence of PRs has been observed in one-third cases of CNS tuberculosis that manifest PRs [5]. Whether PRs cause clinical problems is probably dependent on their size and the anatomical location of the tuberculomas and exudates [2, 5, 15]. Based on these facts, we assume that our patient silently repeated subclinical relapse and remission of PRs, and it has been 10 years since she experienced symptoms of PRs. In addition, there is a possibility that the presence of the VP shunt alleviated the symptoms by adjusting the intracranial pressure. As far as we could research, to date, only two cases of PRs of CNS tuberculosis after the completion of the anti-TB treatment have been reported, and both used an inserted VP shunt to treat hydrocephalus [16]. The patency of the VP shunt in our patient was checked regularly, which prevented hydrocephalus and could have fortunately masked the symptoms even when the tuberculomas obstructed the CSF flow. Thus, we speculate that PRs took a longer time to become apparent.

Whether a lesion is a relapse of CNS tuberculosis or post-treatment PRs remains a clinical challenge when a new tuberculoma or the enlargement of pre-existing tuberculomas is detected after the completion of tuberculous meningitis treatment. Notably, our patient also had asymptomatic residual tuberculomas in the basal cistern. Thwaites et al. reported that 50% of those who demonstrated a complete recovery from tuberculous meningitis reveal asymptomatic tuberculomas on MRI after 270 days of the treatment [15]. Although no consensus guidelines exist to indicate the management of asymptomatic remnant tuberculomas, we should judiciously trace it by MRI for early intervention.

Although the treatment of post-treatment PRs remains to be established, the use of corticosteroids or surgical resection is a choice for treatment. Anecdotal evidence has suggested that the use of corticosteroids reduces symptoms and inflammation in approximately 50% of patients with PRs of CNS tuberculosis [6]. A majority of previous reports have suggested that the lesion of post-treatment PRs is sterile and culture-negative and have proposed that additional anti-TB treatment might not be essential in late-onset PRs [1, 17]. Overall, corticosteroids appear to be safe, at least when the isolated *M. tuberculosis* is susceptible to the first-line anti-TB therapy and when given appropriately for a certain specified period. However, some severe cases are refractory to corticosteroids, and symptoms of PRs persist and worsen. In such cases, alternative anti-inflammatory agents have been tried, and some case reports suggest the use of thalidomide [18, 19], tissue necrosis factor-α antagonists [20, 21], and interferon-γ [22] for PRs resistant to corticosteroids. Nevertheless, further studies are warranted to elucidate the effective therapy and mechanism of post-treatment PRs.

In summary, clinicians should be aware that PRs can occur long after the completion of tuberculous meningitis treatment, thereby necessitating a precise diagnosis with MRI for the early detection of post-treatment paradoxical tuberculomas. In such cases, corticosteroids remain a choice for treatment of post-treatment PRs and are seemingly safe, at least when the isolated *M. tuberculosis* is susceptible to the first-line anti-TB therapy and when given sufficiently for a specified period.

## Abbreviations

Anti-TB: Anti-tuberculosis
CNS: Central nervous system
CSF: Cerebrospinal fluid
FLAIR: Fluid-attenuated inversion recovery
MRI: Magnetic resonance imaging
PCR: Polymerase chain reaction
PR: Paradoxical reactions
VP shunt: Ventriculoperitoneal shunt
